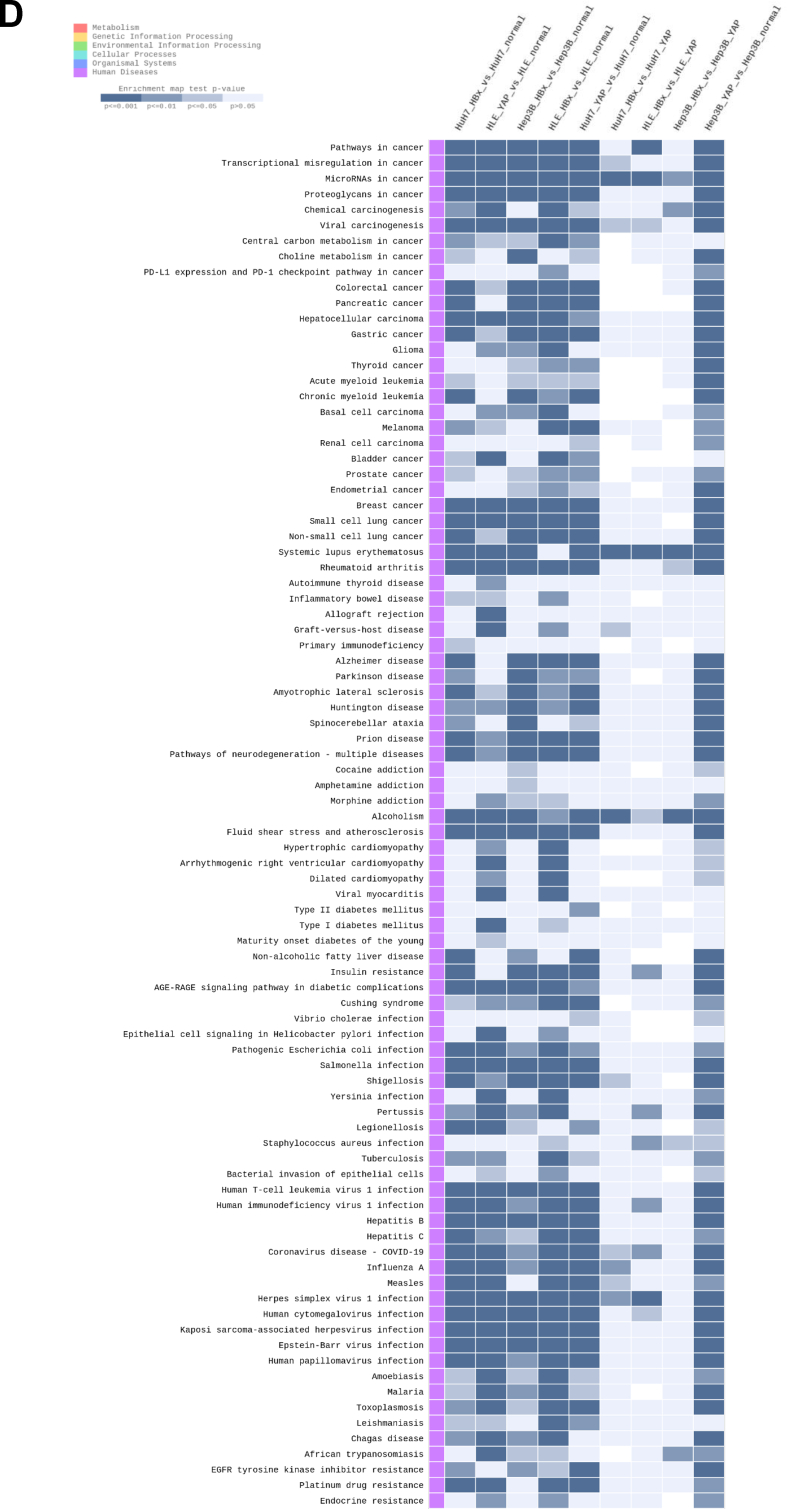# HBx and YAP expression could promote tumor development and progression in HBV-related hepatocellular carcinoma

**DOI:** 10.1016/j.bbrep.2022.101352

**Published:** 2022-09-20

**Authors:** Chiyumi Oda, Kenya Kamimura, Osamu Shibata, Shinichi Morita, Yuto Tanaka, Toru Setsu, Hiroyuki Abe, Takeshi Yokoo, Akira Sakamaki, Hiroteru Kamimura, Satoshi Kofuji, Toshifumi Wakai, Hiroshi Nishina, Shuji Terai

**Affiliations:** a Division of Gastroenterology and Hepatology, Graduate School of Medical and Dental Sciences, Niigata University, Niigata, Niigata, Japan; b Department of General Medicine, Niigata University School of Medicine, Niigata, Niigata, Japan; c Department of Developmental and Regenerative Biology, Medical Research Institute, Tokyo Medical and Dental University, Bunkyo-ku, Tokyo, Japan; d Division of Digestive and General Surgery, Graduate School of Medical and Dental Sciences, Niigata University, Niigata, Niigata, Japan

**Keywords:** HBV, HBx, YAP, Hepatocellular carcinoma

## Abstract

**Background:**

Hepatitis B virus (HBV)-related hepatocellular carcinoma (HCC) accounts for 10%–20% of the total HCC numbers. Its clinical features include the occurrence in the younger generation, large tumors, and poor prognosis. The contribution of hepatitis B virus X (HBx) protein in hepatocytes during activation of various oncogenic pathways has been reported. We aimed to assess the possible association between HBx and Yes-associated protein (YAP) expression in the liver tissue and the clinical features of HBV-related HCC.

**Methods:**

The relationship between HBx and YAP expression was examined *in vivo* using HCC tumor and peritumor tissues (n = 55). The clinical information including tumor size, marker, and the prognosis was assessed with protein expressions. The *in vitro* gene expression analyses were conducted using HBx- and YAP-overexpressing HCC cell lines.

**Results:**

Among 19 cases of HBV-related, 17 cases of hepatitis C virus (HCV)-related, and 19 cases of nonviral-related HCC, the HBV-related tumor showed the largest size. The HBx-stained area in the tumor and peritumor tissue showed a significant correlation with tumor size and serum α-fetoprotein level. YAP expression was higher in HBV-related tumor tissue than in the peritumor tissue and HCV-related tumor. Additionally, HBx and YAP protein expressions are correlated and both expressions in the tumor contributed to the poor prognosis. An *in vitro* study demonstrated that *HBx* and *YAP* overexpression in the hepatocytes activate the various oncogenic signaling pathways.

**Conclusions:**

Our study demonstrated that YAP expression in the liver of HBV-infected patients might be the key factor in HBV-related HCC development and control of tumor-related features.

## Highlights

• HBx and YAP expression are related to the HCC size and tumor marker level.
• HBx activates YAP expression and both protein expressions are correlated.
• Co-expression of HBx and YAP contributes to the poor prognosis of HCC cases.
• HBx and YAP expression *in vitro* activated various oncogenic pathways.

## 1 Introduction

In 2020, liver cancer was the sixth most commonly diagnosed cancer and the third leading cause of cancer-related deaths worldwide [1,2]. More than 90% of liver cancers are hepatocellular carcinoma (HCC) and its etiologies include hepatitis C virus (HCV)-related liver disease, hepatitis B virus (HBV)-related liver disease, alcohol abuse, and nonalcoholic fatty liver disease [[1], [2], [3], [4]]. HBV-related HCC accounts for 10%–20% of total HCC numbers and have shown no changes since the 1990s [5]. The clinical features of HBV-related HCC include the occurrence in younger generation, large tumors (>5 cm), and poorer survival when compared with HCV-related HCC [6].

These features are related to various factors including chronic inflammation, the integration of HBV-DNA into the host hepatocyte genomic DNA, and the expression of viral proteins, which transactivate human oncogenes [[6], [7], [8], [9]]. The molecular mechanisms of HBV-related HCC occurrence include the contribution of hepatitis B virus X (HBx) protein, which consists of 154 amino acids and is essential for the effective replication of HBV. HBx associates with several host protein expressions in the oncogenic signaling pathways including cell cycle-related proteins, DNA damage response-proteins, apoptosis-related proteins, p53, Wnt-β-catenin, JAK/STAT, RAS pathways, and Hippo signaling pathways [[10], [11], [12]]. Yes-associated protein (YAP) is a member of the Hippo signaling pathway and is a transcriptional coactivator that promotes tissue growth by promoting cell proliferation and inhibiting apoptosis. YAP activates various oncogenic pathways when retained in the nucleus. Once it moves out to the cytoplasm and is phosphorylated, its activity is suppressed. The Hippo signaling pathway manages its activity and when the pathway is inhibited, YAP continuously remains in the nucleus and shows oncogenic activity [[13], [14], [15], [16], [17]].

Indeed, we have shown that YAP overexpressed liver in wild-type mice showed the development of huge HCC in the short term and the tumors were heterogeneous, histologically [18]. Interestingly, HBx transgenic mice increase YAP protein expression postnatally in the hepatocytes, and YAP accumulation in the nucleus resulted in the hepatocytes’ carcinogenesis [12]. Therefore, it is reasonable to hypothesize that the HBx-YAP axis is involved in the HBV-related HCC occurrence and development of huge tumors and poor prognosis in HBV-related HCC [18]. Since there are no reports assessing the HBx and YAP expression patterns in HCC tumor and peritumor tissue with clinical information, we have assessed the HBx and YAP expression in human HBV-related HCC samples and their association with tumor size, histological findings, serum tumor markers, and prognosis, comparing them with HCC related to other etiologies.

## 2 Material and method

### 2.1 Ethical considerations

The study protocol was approved by the Ethics Committee and Institutional Review Board of Niigata University School of Medicine (Nos. 751-716 and G2018-0023). Informed written consent was obtained from each participant. The study was conducted following the standards of the 2013 Declaration of Helsinki. The final manuscript was reviewed and approved by all the authors.

### 2.2 Histological analysis

The required tissue samples were obtained from the surgically excised HCC tumor tissues in Niigata University Hospital. Hematoxylin and eosin were used to stain the HCC tumor cells and the surrounding hepatic tissues in addition to the immunohistochemical staining conducted with an anti-YAP antibody (No. 4912, Cell Signaling Technology) using 1:100 dilution and with an anti-HBx antibody (ab39716, Abcam) at 1:500 dilution using the Vectastain Elite ABC rabbit IgG kit (PK-6101, Vector Laboratories, Burlingame, CA, USA) and 3,3′-diaminobenzidine chromogen tablets (Muto Pure Chemicals, Tokyo, Japan). Then, the images were randomly captured from each tissue section, and quantitative analysis was conducted using ImageJ software (version 1.6.0_20; National Institutes of Health, Bethesda, MD, USA) with RGB-based protocol as reported previously [19].

### 2.3 Cells

Human hepatoma HLE and HepG2 cell lines were purchased from the Japanese Collection of Research Bioresources Cell Bank (National Institutes of Biomedical Innovation, Health and Nutrition, Ibaraki, Osaka) and Hep3B2.1-7 (HB-8064) was purchased from American Type Culture Collection (ATCC, Manassas, VA). A minimum Essential Medium containing 10% fetal bovine serum and 100 U/mL of penicillin and streptomycin was used for cell culture. A 5% CO_2_ humidified incubator at 37°C was used for cell incubation.

### 2.4 Plasmids

The YAP-expressing plasmid was constructed using the full-length complementary DNA of human YAP ligated into XbaI restriction sites of the expression vector of the pLIVE vector (Mirus Bio., Madison, WI, USA). The HBx-expressing plasmid was constructed using the full-length complementary DNA of HBx cloned in the pCMV6-Entry Tagged vector and was kindly provided by Sei Kakinuma and Mamoru Watanabe at Tokyo Medical and Dental University. Either mock, YAP-expressing, or HBx-expressing plasmid were transfected into HLE, HepG2, and Hep3B cells using FuGENE HD Transfection Reagent (Promega, Madison, WI, USA) following the instruction and harvested at the appropriate time points. The Plasmid Mega Kit (Qiagen, Hilde, Germany) was used for plasmid purification.

### 2.5 Whole transcript expression arrays and bioinformatics analyses

Affymetrix mRNA expression analysis was performed to investigate the different gene expression profiles and to perform gene annotation on a set of useful genes by Macrogen Japan Corp. (Koto City, Tokyo, Japan). Detailed information was shown in **Information of the Supplementary Materials**.)

### 2.6 Cell growth assay

Cells were placed in 96-well culture dishes, 2 × 10^4^ cells per well, in 100 μL of the medium. After treatment and at the indicated time, water-soluble tetrazolium salt (WST) reagents were added to the cells. Then, Premix WST-1 Cell Proliferation Assay System (Takara Inc., Kyoto, Japan) was used for cell counting.

### 2.7 Statistical analyses

The obtained data were analyzed using the paired *t-*test and categorical variables were analyzed by the Mann-Whitney-Wilcoxon test. The relationship between HBx and YAP positively stained area, tumor size, and serum α-fetoprotein (AFP) level were analyzed using Pearson's correlation test. *p* < 0.05 was considered statistically significant. Graphpad Prism 9 software (version 9.3.1; GraphPad, San Diego, CA, USA) was used for the analyses and *p* < 0.05 was considered statistically significant.

## 3 Results

### 3.1 Patient characteristics

To examine the effect of HBx and YAP on HCC development, the liver tissue of patients treated with surgical resection with no prior chemotherapy or chemoembolization was subjected to histological analyses. In total, 54 cases of stage I HCC, according to tumor–node–metastasis Classification of the American Joint Committee on Cancer for HCC, who underwent surgical resection between 2012 and 2019, including 19 cases of HBV-related HCC, 17 cases of HCV-related HCC, and 19 cases of nonviral-related HCC were assessed. The nonviral-related HCC group includes six cases of nonalcoholic steatohepatitis and 13 cases of alcoholic liver injury. The baseline characteristics of the patients showed no statistical differences between the three groups for age, gender, presence of liver cirrhosis, Child–Pugh grade, serum AFP level, and histological classification (Table 1). Tumor size was significantly larger in the HBV-related HCC subgroup than in the HCV-related subgroup (median 35.0 mm vs 21 mm, respectively) (*p* = 0.06) (Table 1).

**Table 1:** Characteristics of the patients.

Characteristic	Group			MWW test *P*-value
	HBV	HCV	Non-viral	
	*n* = 19	*n* = 17	*n* = 19 (NASH, 6; Alcoholic, 13)	
Age (yr)				0.67
Median	64.0	70.0	68.0	
Range	46–81	45–80	51–79	
Gender				0.62
Female	4	5	3	
Male	15	12	16	
Cirrhosis				0.77
Yes/no	5/14	7/10	8/11	
Child–Pugh grade				0.37
A/B/C	19/0/0	16/1/0	17/2/0	
Tumor Size (mm)				0.06
Median	35.0	21.0	31.0	
Range	8.0–130.0	14.0–90.0	10.0–120.0	
AFP (ng/mL)				0.27
Median	6.0	8.4	8.0	
Range	2.0–24434.0	3.0–292.2	2.0–10817.0	
Histology (differentiation)				0.22
well-mod. HCC	14	16	12	
poor HCC	5	1	7	

MWW, Mann–Whitney–Wilcoxon test; HBV, hepatitis B virus; HCV, hepatitis C virus; AFP, alpha-Fetoprotein; HCC, hepatocellular carcinoma; mod., moderately.

### 3.2 HBx and YAP protein expression and HCC

Immunohistochemical analyses were conducted on the HBV-related tumor and its surrounding tissue (peritumor) using an anti-HBx and anti-YAP antibodies (Fig. 1). Supplementary Table S1 summarizes the pattern of HBx staining (Fig. 1A) in HCC tumor and peritumor tissue. In HBV-related HCC, 63% of cases (n = 12 out of 19 cases) stained positive (>5%) for HBx in tumor and/or peritumor tissues. The level of HBx-stained area showed 9.8% ± 7.2% and 7.7% ± 3.7% in peritumor and tumor tissues in these 12 cases with no significant differences (Fig. 1B). The HCV-related HCC and nonviral-related HCC showed no meaningful positively stained cells for HBx as it was expected (Fig. 1B). In HBV-related HCC, the HBx-stained area showed a significant correlation with both the tumor size (Fig. 1C) and the serum AFP level (Fig. 1D). The HBV-related HCC showed that 84% of cases (n = 16 of 19 cases) were stained positively for YAP in tumor and/or nontumor tissues (Fig. 1E). The level of staining showed significant differences of 4.9% ± 0.5% and 19.3% ± 2.5% in peritumor and tumor tissues in HBV-related HCC cases (Fig. 1F). HCV-related HCC showed that 71% of cases (n = 12 of 17 cases) were stained positively for peritumor and/or tumor tissues and the level of staining showed 2.6% ± 0.2% and 8.1% ± 1.3% in peritumor and tumor tissues in HCV-related HCC cases which was significantly higher in the tumor tissues but lower than HBV-related HCC (Fig. 1F). Nonviral-related HCC cases showed that 80% of cases were positively stained for YAP (n = 15 of 19 cases); however, no differences were seen between the peritumor and tumor tissues. The YAP-stained area and HCC tumor size showed a significant correlation (*p* < 0.05, Fig. 1G); although serum AFP level also showed a tendency to correlate with the YAP-stained area, it was not statistically significant (Fig. 1H). These results suggest that HBx expression in the liver is related to the progression of the tumor size and YAP expression is related to carcinogenesis in the viral-induced HCC, especially in HBV-related HCC.

**Fig. 1:**
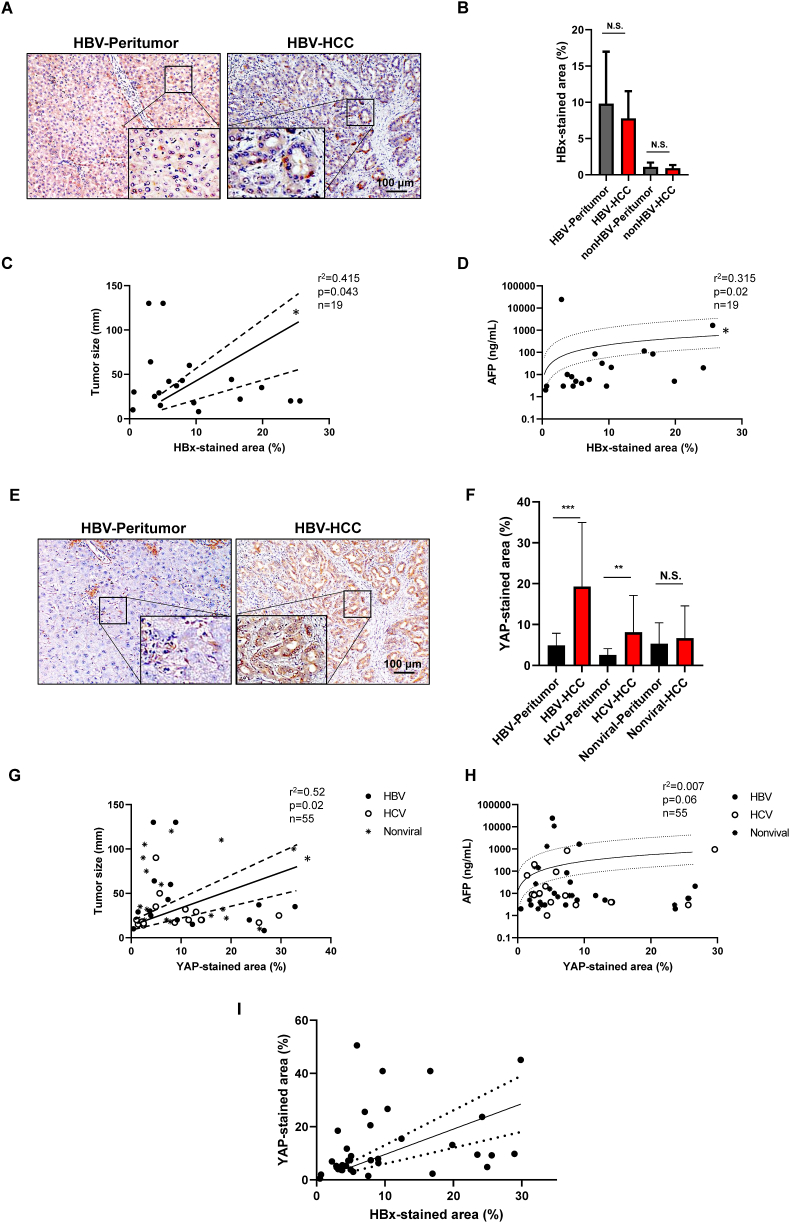
HBx and YAP protein expression in HCC progression (A) Representative HBx staining in HBV-related HCC tumor and its peritumor tissue in the human liver. The scale bar represents 100 μm. (B) Quantitative analysis of the HBx-stained area in HBV-related HCC tumor (HBV-HCC), its peritumor tissue (HBV-Peritumor), non-HBV-related HCC tumor (non-HBV-HCC), and its peritumor tissue (non-HBV-Peritumor). The values represent mean ± SD (every two samples from the tissue, n = 38 for each group in HBV-related and n = 72 for each group in non-HBV-related). N.S., not significant. Student's *t*-test. (C) Relationship between HBx-stained area and tumor size in HBV-related HCC tumor (HBx-stained values are average of every two samples from the tissue, n = 19). *p < 0.05, Pearson's correlation test. r, correlation coefficient. Continuous lines are the best hit lines accompanied by dotted lines showing 95% confidence intervals. (D) Relationship between HBx-stained area and serum α-fetoprotein (AFP) level (HBx-stained values are average of every two samples from the tissue, n = 19). *p < 0.05, Pearson's correlation test. r, correlation coefficient. Continuous lines are the best hit lines accompanied by dotted lines showing 95% confidence intervals. (E) Representative YAP staining in HBV-related HCC tumor and its peritumor tissue in the human liver. The scale bar represents 100 μm. (F) Quantitative analysis of the YAP-stained area in HBV-HCC, HBV-Peritumor, HCV-related HCC tumor (HCV-HCC), its peritumor tissue (HCV-Peritumor), nonviral-related HCC tumor (Nonviral-HCC), and its peritumor tissue (Nonviral-Peritumor). The values represent mean ± SD (every two samples from the tissue, n = 38 for each group in HBV-related, n = 34 for each group in HCV-related, and n = 38 for each group in nonviral-related). **p < 0.01 and ***p < 0.001, N.S., not significant. Student's *t*-test. (G) Relationship between YAP-stained area and tumor size. YAP-stained values are the average of every two samples from the tissue, n = 19, 17, and 19 for HBV-, HCV-, and nonviral-related HCC tumors, respectively. *p < 0.05, Pearson's correlation test. r, correlation coefficient. Continuous lines are the best hit lines accompanied by dotted lines showing 95% confidence intervals. (H) Relationship between HBx-stained area and serum α-fetoprotein (AFP) level. Pearson's correlation test. r, correlation coefficient. Continuous lines are the best hit lines accompanied by dotted lines showing 95% confidence intervals. (I) Relationship between HBx- and YAP-stained area. Every two samples from 19 tissues of HBV-related HCC were assessed. ****p < 0.0001, Pearson's correlation test. r, correlation coefficient.

### 3.3 HBx and YAP expression

To determine the relationship between HBx and YAP expression in HBV-related HCC, the co-expression of these proteins was assessed in HCC tumor tissues (Fig. 1I). Among the 19 HBV-related HCC tissues, 12 cases (63%) were positively stained (>5%) for both HBx and YAP, four cases were positive for YAP, and three cases showed negative for both protein expressions. No cases were positive only for HBx protein in the tumor tissue. Table 2 summarizes the clinical information of these 19 cases. The tumors negative for both HBx and YAP (n = 3) tended to be well to moderately differentiated HCC (100%) and showed better prognosis (PFS) than cases positive for both proteins and YAP-only positive cases. For the YAP-only positive cases (n = 4), three cases were well-differentiated tumors (75%) with PFS of 615 days and one case of poorly differentiated tumors (25%) with PFS of 363 days. Among the 12 cases of both proteins positivity, eight were well-differentiated tumors (75%) with PFS of 539 days and four cases were poorly differentiated tumors (25%) with PFS of 64 days. These results indicate that YAP expression in HBx expressed liver might be one of the key factors of HBV-related HCC development and double positive expressions would be related to a higher malignant potentiality.

**Table 2:** HBx and YAP staining and tumor characteristics.

Case	n = 12	n = 0	n = 4	n = 3
HBx (+, >5%)	+	+	–	–
YAP (+, >5%)	+	–	+	–
Histology				
well-mod. HCC	8	0	3	3
PFS (Days, median) (Days, range)	539.0 22–2561	N.A.	615.0 226–1787	1100.0 195–2212
poor HCC	4	0	1	0
PFS (Days, median) (Days, range)	64.0 20–269	N.A.	363 N.A.	N.A.

PFS, progression-free survival; N.A., not applicable; HCC, hepatocellular carcinoma; mod., moderately.

### 3.4 Gene expression modification in HBx and YAP overexpressed cells

To determine the effect of YAP expression in HBV-positive cells on gene expression modification in the liver cells, the mRNA expression analyses of HBx and YAP overexpressed HCC cell lines were performed by producing HBx-overexpressing, YAP-overexpressing, and HBx + YAP-overexpressing cell lines by transfecting plasmid DNA expressing HBx and YAP genes into hepatoma cell lines of HLE, HuH-7, and Hep3B.

The GO enrichment analysis of common DEGs based on the mRNA expression of HBx-overexpressing cell lines showed that common DEGs in a cellular component (CC), molecular functions (MFs), and biological process (BP) terms include the nuclear proteins including complex for DNA replication, receptor–ligand activity, growth activity, DNA binding, and DNA replication origin binding, epithelial cell proliferation, cell cycle, DNA replication-dependent nucleosome assembly, and DNA replication (Supplementary Fig. S1). The YAP-overexpressing cell lines showed that common DEGs include DNA packaging complex, nuclear proteins including complex for DNA replication, damaged DNA binding activity, cell cycle process, DNA replication, cell cycle checkpoint, and cell cycle G1/S phase transition activity (Supplementary Fig. S2). The Kyoto Encyclopedia of Genes and Genomes (KEGG) pathway analysis (Fig. 2 and Supplementary Fig. S3) indicated activation of metabolic pathways, viral infection-related pathways, pathways in cancer, MAPK, PI3K-Akt signaling pathway, cell cycle pathway, cellular senescence pathway, and DNA replication pathways in HBV- and YAP-overexpressed cell groups compared with the mock-transfected cells (Fig. 2A and B). Additionally, HBV-overexpressed cells showed involvement of the Hippo signaling pathway in its pathology (Supplementary Fig. S3A). Moreover, YAP-overexpressed cell lines showed higher activation of MAPK, NF-κB, RAS, and TNF signaling pathway-related genes than HBx-overexpressed cells (Fig. 2B). These results suggest that HBx and YAP overexpression in the hepatocytes may activate the cell proliferation, HBx induced YAP activation, and YAP expression may further activate the changes.

**Fig. 2:**
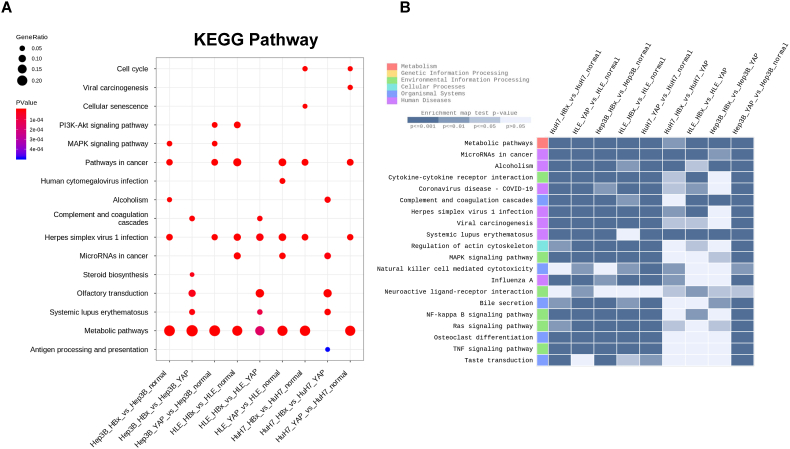
(A) Dot plot of KEGG pathway (B) Heatmap of KEGG enrichment analysis.

## 4 Discussion

As there are no yet approved drugs to eradicate HBV from the hepatocytes of HBV-induced chronic hepatitis patients, it is essential to understand the pathogenesis of HBV-related HCC. Molecular biology research revealed various genetic and epigenetic oncogenic alterations in the step-by-step HCC development. These genes are involved in the oncogenic pathways of cell cycle regulation, Wnt-β-catenin, JAK/STAT, RAS, TGF-β, etc. [1,2]. HBx [20,21] and YAP [22,23] proteins have been reported to be involved in HBV-related HCC occurrence [12]. However, the expression patterns in HCC and peritumor tissue, and their relationship with clinical information including tumor characteristics, tumor marker, and prognosis have not been examined.

Our study demonstrated that HBx protein expression is related to YAP expression and tumor progression in human liver tissue. Moreover, following YAP expression is related to the HBV-related HCC occurrence based on the clinical information and histological analyses. HBx and YAP expression showed a significant association, and in addition, tumors expressing both proteins showed significantly poorer prognosis. These results suggest that YAP expression in HBx expressing liver might be one of the key factors of HBV-related HCC development, its size, tumor marker level, and poor prognosis.

The results are supported by the fact that HBx transgenic mice show the activation of JAK/STAT, Wnt-beta catenin, YAP pathway, and accumulation of DNA damage in the hepatocytes, which led to HCC occurrence [10,24]. Additionally, YAP overexpression results in hepatocarcinogenesis within a short period in wild-type mice [18] and was associated with poorer tumor differentiation, high serum AFP level, and poor prognosis in humans [13]. Consistent with the *in vitro* study, which demonstrated that HBx protein activates YAP expression by binding to the promoter of YAP [12], HBx transgenic mice increase YAP expression postnatally and its accumulation in the nucleus shows a carcinogenesis effect [12,25]. Additionally, a significant relationship between *HBx* and *YAP* mRNA levels in 33 HCC tissues was reported [12]. These results support our data, which suggest that YAP expression in HBx expressing liver might be one of the key factors of HBV-related HCC development and poor prognosis. Additionally, our previous study that proves the effective therapeutic effect of YAP-targeted gene therapy on HCC prevention [18] further supports these results. The gene expression analyses of HBx and/or YAP-overexpressing cell lines that showed the activation of cell proliferation-related MAPK, NF-κB, RAS, and TNF signaling pathways, further supported our results.

The limitations of our study involve the small sample size and the molecular mechanisms of YAP expression in HBx positive hepatocytes on the tumor size and progression; further studies should include cell-based phenotypic analyses by single-cell assays and HBx and YAP gene transfer and molecular mechanisms assessment for the hepatocarcinogenesis *in vivo*.

In summary, YAP expression in the liver of HBV-infected patients might be a key factor in HBV-related HCC development and a therapeutic target for human HCC.

## Funding

The research was supported in part by a Grant from Japan agency for Medical Research and Development JP18fk0210042, JP19fk0210042, and JP20fk0210042 to Nishina H and Terai S, and JP22fk0310508h to Nishina H, Terai S, and Kamimura K.

## Author contributions

CO, KK, HN, and ST contributed to the study conception and design. Material preparation, data collection, and analysis were performed by CO, KK, OS, SM, YT, TS, HA, TY, AS, HK, and ST. The first draft of the manuscript was written by CO, KK, and ST, and all authors read and approved the final manuscript.

## Declaration of competing interest

The authors declare that they have no known competing financial interests or personal relationships that could have appeared to influence the work reported in this paper.

## Figures and Tables

**figs1:**
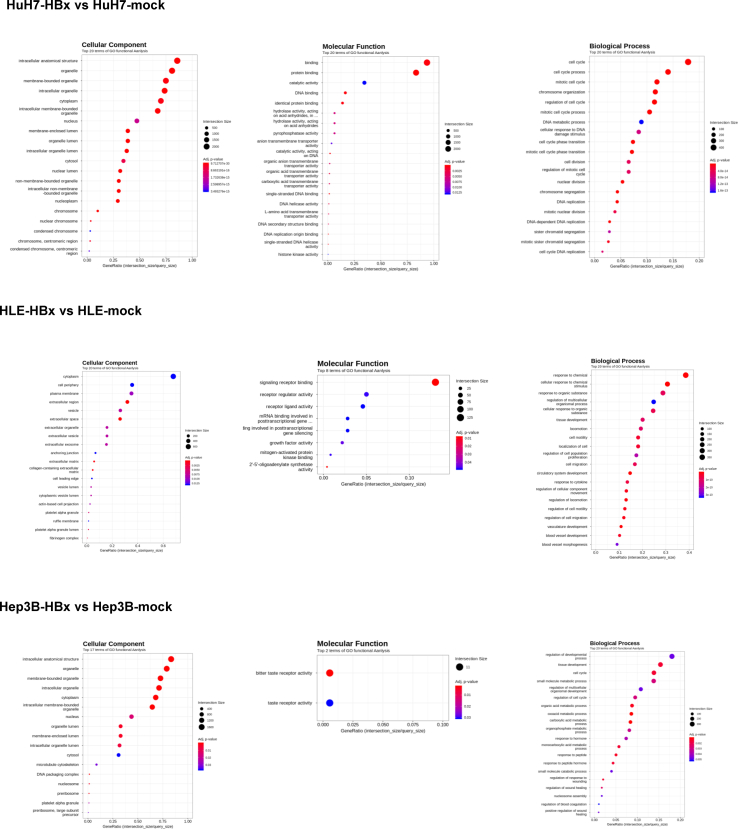


**figs2:**
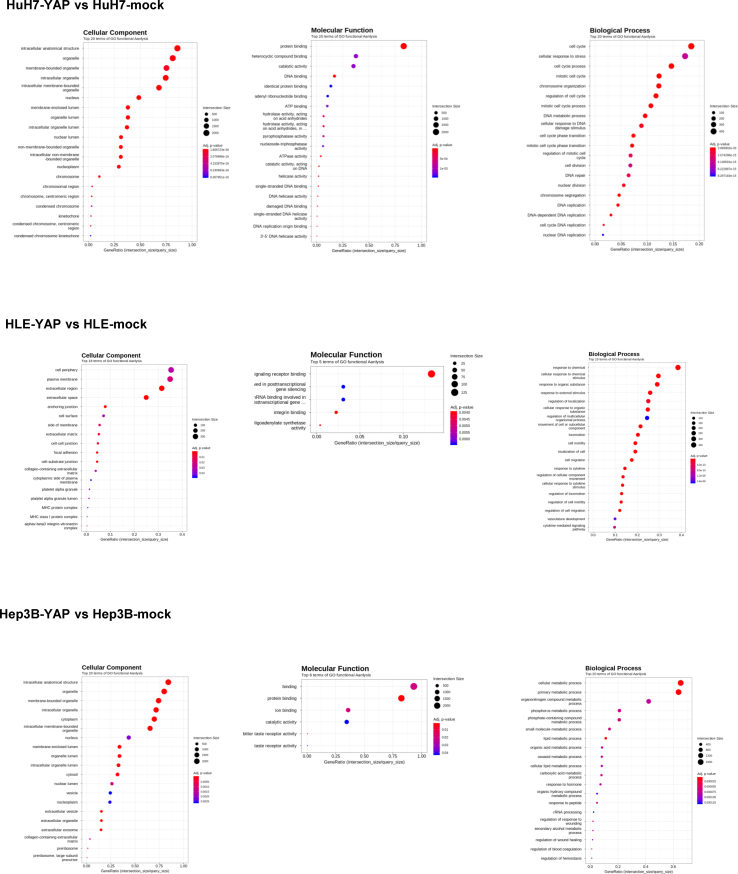


**figs3:**
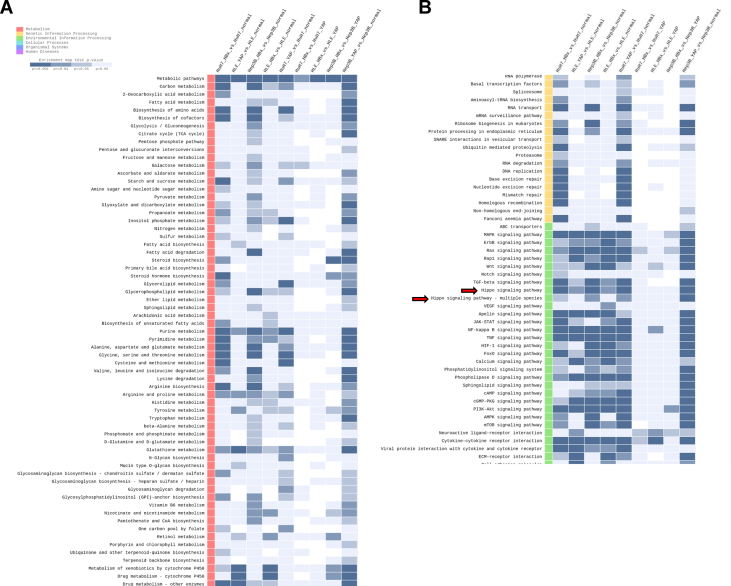


**figs4:**
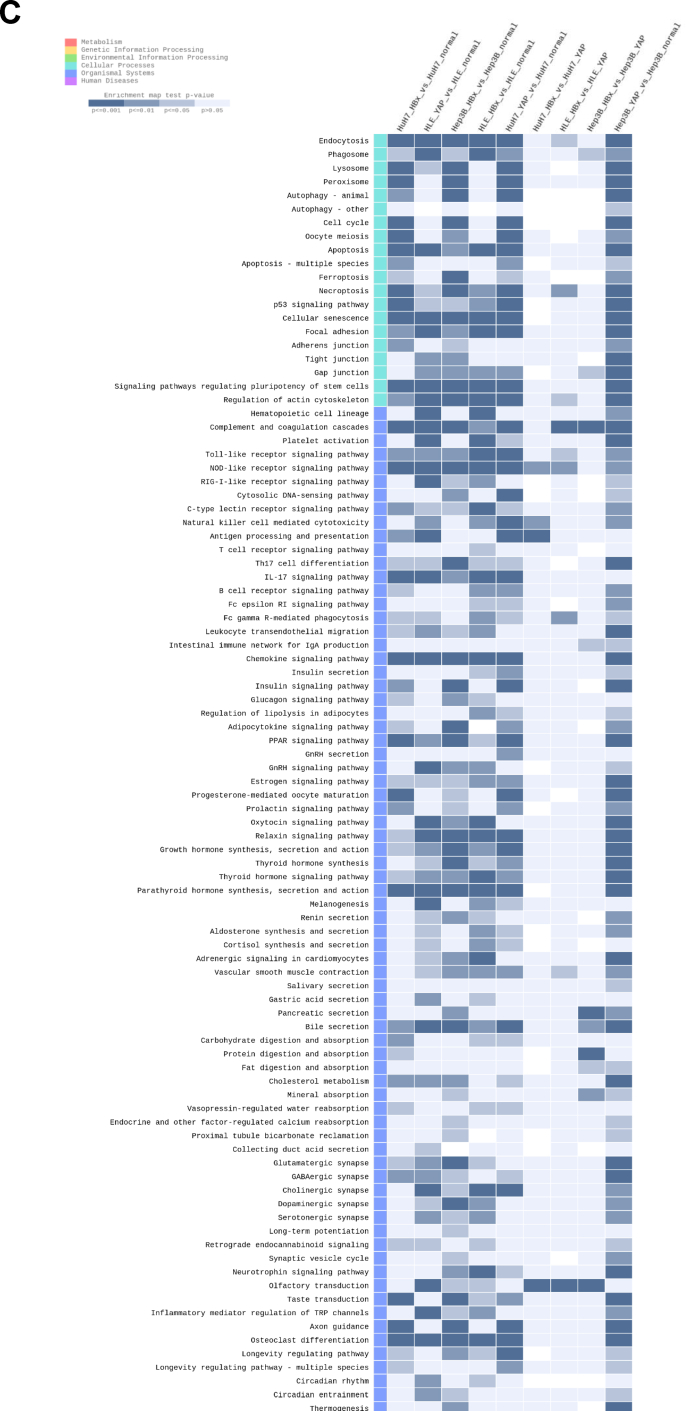


**figs5:**