# Supplementary material for: HBx and YAP expression could promote tumor development and progression in HBV-related hepatocellular carcinoma
Source: Biochem Biophys Rep. 2022 Sep 20;32:101352. doi: 10.1016/j.bbrep.2022.101352 (PMC9490549; doi:10.1016/j.bbrep.2022.101352)
Supplement: Multimedia component 1 [file mmc1.docx]

**Information of the Supplementary Materials**

**Materials and Methods for Supplementary Materials**

***Whole transcript expression arrays and bioinformatics analyses***

RNA was purified from the cells and the purity and integrity were evaluated by ND-1000 Spectrophotometer (NanoDrop, Wilmington, USA), Agilent 2100 Bioanalyzer (Agilent Technologies, Palo Alto, USA). The Affymetrix whole transcript expression array process was executed according to the manufacturer's protocol (GeneChip Whole Transcript PLUS reagent Kit). Approximately 5.5 μg of labeled DNA target was hybridized to the Affymetrix GeneChip at 45°C for 16 hours. Hybridized arrays were washed and stained on a GeneChip Fluidics Station 450 and scanned on a GCS3000 Scanner (Affymetrix). Signal values were computed using the Affymetrix® GeneChip™ Command Console software. The robust multi-average (RMA) method implemented in Affymetrix® Power Tools was used to summarize and normalize the data. The results were exported with gene-level RMA analysis and the differentially expressed gene (DEG) analysis was done. Fold change was used to determine the statistical significance of the expression data. For a DEG set, as a measure of similarity, the Hierarchical cluster analysis was performed using complete linkage and Euclidean distance. Gene Ontology (GO) (<http://geneontology.org>) and KEGG (<http://kegg.jp>) were used for gene-enrichment and functional annotation analysis for a significant probe list. All data analysis and visualization of differentially expressed genes was conducted using R 3.3.3 ([www.r-project.org](http://www.r-project.org)).

**Legends for Supplementary Figures**

**Supplementary Figure S1.**

Gene set enrichment analysis per cellular component, molecular function, and biological process in HuH7-HBx vs HuH7-mock, HLE-HBx vs HLE-mock, and Hep3B-HBx vs Hep3B-mock.

**Supplementary Figure S2.**

Gene set enrichment analysis per cellular component, molecular function, and biological process in HuH7-YAP vs HuH7-mock, HLE-YAP vs HLE-mock, and Hep3B-YAP vs Hep3B-mock.

**Supplementary Figure S3.**

KEGG enrichment heatmap per: **(A)** metabolism; **(B)** genetic information processing and environmental information processing; **(C)** cellular processes and organismal systems; and **(D)** human diseases.

**Supplementary Table S1. HBx positively stained area**
