# Supplementary material for: HBx and YAP expression could promote tumor development and progression in HBV-related hepatocellular carcinoma
Source: Biochem Biophys Rep. 2022 Sep 20;32:101352. doi: 10.1016/j.bbrep.2022.101352 (PMC9490549; doi:10.1016/j.bbrep.2022.101352)
Supplement: Multimedia component 2 [file mmc2.docx]

**Supplementary Table S1. HBx positively stained area**

| **HBx staining (+, >5%)** | **n=10** | **n=2** | **n=1** | **n=6** |
| --- | --- | --- | --- | --- |
| **Tumor** | **+** | **+** | **-** | **-** |
| **Non-tumor** | **+** | **-** | **+** | **-** |
